# Nonlinear Analysis of Electroencephalogram in Schizophrenia Patients with Persistent Auditory Hallucination

**DOI:** 10.4306/pi.2008.5.2.115

**Published:** 2008-06-30

**Authors:** Seung-Hwan Lee, Jung-Suk Choo, Wu-Young Im, Jeong-Ho Chae

**Affiliations:** 1 Department of Psychiatry, College of Medicine, Inje University, Ilsanpaik Hospital, Goyang, Korea.; 2 Clinical Emotion and Cognition Research Laboratory, Goyang, Korea.; 3 Department of Psychiatry, St. Mary's Hospital, College of Medicine, The Catholic University of Korea, Seoul, Korea.

**Keywords:** Auditory hallucination, Schizophrenia, Correlation dimension, Dimensional complexity, Gamma and beta frequency oscillations, Electroencephalogram

## Abstract

**Objective:**

The recent nonlinear analyses of electroencephalogram (EEG) data have shown that the correlation dimension (D2) reflects the degree of integration of information processing in the brain. There is now considerable evidence that auditory hallucination (AH) reflects dysfunctional gamma and beta frequency oscillations. Gamma oscillations are thought to reflect internally driven representations of objects, and the occurrence of subsequent beta oscillations can reflect the modification of the neuronal circuitry used to encode the sensory perception. The purpose of this study was to test whether AH in schizophrenia patients is reflected in abnormalities in D2 in their EEG, especially in the gamma and beta frequency bands.

**Methods:**

Twenty-five schizophrenia patients with a history of treatment-refractory AH over at least the past 2 years, and 23 schizophrenia patients with no AH (N-AH) within the past 2 years were recruited for the study. Artifact-free 30-s EEG epochs during rest were examined for D2.

**Results:**

The AH patients showed significantly increased gamma frequency D2 in Fp2 and decreased beta frequency D2 in the P3 region compared with the N-AH patients. These results imply that gamma frequency D2 in the right prefrontal cortex is more chaotic and that beta frequency D2 in the left parietal cortex is more coherent (less chaotic) in AH patients than in N-AH patients.

**Conclusion:**

Our study supports the previous evidence indicating that gamma and beta oscillations are pivotal to AH, and also shows the distinctive dimensional complexity between the right prefrontal and left parietal cortexes as the underlying biological correlates of AH in schizophrenia patients.

## Introduction

There have been several reports that high-frequency oscillations (especially beta) are more predominant in the electroencephalogram (EEG) in schizophrenia patients with auditory hallucination (AH) than in control subjects.^1^,^2^ The gamma to beta transition in response to novel auditory stimuli has been observed in human EEG^3^ as well as in animal studies.^4^,^5^ Furthermore, there is evidence of a strong correlation between the gamma and beta frequency bands in response to novel auditory stimuli.^6^-^8^ Thus, the transition from gamma to beta oscillations is believed to be crucial to auditory sensory perception.

Functional neuroimaging studies for schizophrenia patients during AH have revealed activation in speech-related areas such as the left superior temporal cortex,^9^,^10^ left inferior parietal cortex,^9^,^11^ and left inferior frontal cortex.^12^ In addition, the activation of the right superior temporal cortex,^13^ thalamus and cingulate has been shown to occur during AH.^14^,^15^ One other study of AH revealed the activation of the frontal cortex, amygdala and hippocampus in addition to that of the primary auditory cortex.^16^ These findings suggest that AHs are a complex feature of psychosis that reflects abnormal activities in multiple interrelated regions.

Moreover, there is accumulating evidence of functional differences among these brain regions. For example, whilst the prefrontal cortex is believed to be involved in internally driven sensory representation and executive functions,^17^,^18^ the temporoparietal cortex is involved in the sensorial encoding of sound.^19^

The analysis of EEGs based on nonlinear dynamics provides information on the dimensional complexity (DC), such as the correlation dimension (D2), entropy, and first positive Lyapunov exponent. The D2 of the EEG reflects the complexity (or flexibility) of information processing of the brain, since it is defined as the number of independent variables that are necessary to describe the behavior of a dynamic system. Although research into the DC remains largely insufficient, the complexity might reflect the processing of information in the brain and include both the integration of the activity of functionally segregated neuronal groups and the integration of incoming stimuli with ongoing, spontaneous brain activity.^20^

Several studies have investigated DC in patients with schizophrenia, but the findings have been contradictory, variously indicating increased or decreased DC in these patients. However, a detailed analysis of the disease statuses of the subjects of the previous studies reveals that there is a consistency in the previous research findings. While the studies which reported increased DC usually recruited newly onset, drug naive, and active symptomatic schizophrenia patients,^21^-^24^ those that reported decreased DC recruited relatively chronic, drug-taking, and less symptomatic patients.^25^-^27^ Accordingly, we concluded that it is fruitless to address the DC of schizophrenia patients without considering their disease courses and specific pathologies, which can affect the neuronal activity in their brain.

Our basic hypothesis was that schizophrenia patients with persistent AH would generally show increased D2, but that their D2 value might vary according to the functional activity of the brain region. The purpose of the present study was to elucidate how D2 changes depending on the brain region in schizophrenia patients with AH.

## Methods

### Subjects

Twenty-five schizophrenia patients with a current history of persistent AH over at least the past 2 years were recruited. Ten of the patients were referred from other hospitals following the placement of local advertisements. All of the patients met the Diagnostic and Statistical Manual of Mental Disorders IV (DSM-IV) criteria for schizophrenia based on both the Structured Clinical Interview for DSM-IV and psychiatric chart review.^28^,^29^ Twenty-three schizophrenia patients with no history of AH (N-AH) in the past 2 years were also recruited. The exclusion criteria were a history of central nervous system diseases (e.g., epilepsy or cerebrovascular accident), alcohol or drug abuse, electroconvulsive therapy, mental retardation, head injury with loss of consciousness, or hearing impairment. All of the AH and N-AH patients were right-handed. AH status (i.e., experiencing treatment-refractory AH for the past 2 years) was determined by an Structured Clinical Interview for DSM-IV (SCID) interview and chart histories. To further assess the more-recent AH status, the hallucinatory behavior subscore from the Positive And Negative Syndrome Scale (PANSS) for the 2 months prior to testing was examined.^30^ As can be seen in Table 1, the scores on the hallucinatory behavior subscore were significantly higher in the AH patients than in the N-AH patients.

The AH and N-AH patients were group matched for age, sex, duration of illness, duration on stable medication, number of prior hospitalizations, and PANSS scores (total, positive, and negative scores; Table 1). At the time of enrollment, all of the patients in both groups were on stable antipsychotic regimens of either risperidone (N=34, AH/N-AH=18/16) or olanzapine (N=14, AH/N-AH=7/7) and had not used benzodiazepine for at least 2 weeks. All of the subjects signed a written informed consent approved by the Inje University Ilsanpaik Hospital Institutional Review Board prior to their participation.

### Electroencephalogram recording and analysis

With the subject in a relaxed state, the EEG was recorded with the eyes alternatively closed and open for 1 min each, for a total of 15 min from 18 scalp locations (Fp1, F3, C3, P3, Fp2, F4, C4, P4, F7, T3, T5, O1, F8, T4, T6, O2, T1, and T2) using the international 10-20 system with a linked ear reference. The EEG data were collected using a conventional 32-channel electroencephalograph (Nicolete Biomedical, Madison, WI, USA) in a dimly lit, soundproof, electrically shielded room. Horizontal eye movements were recorded across electrodes 1-cm lateral to the outer canthus of each eye. The EEG was recorded at a rate of 250 Hz, with a sensitivity of 7 µV, and bandpass filtered at 1-70 Hz. Eye-blinking artifacts and segments contaminated by other artifacts on visual inspection were excluded. The resting EEG corresponding to one artifact-free 30-s epoch with the eyes closed were subjected to nonlinear analysis. The band pass filtering was used to extract the beta and gamma frequency bands before the nonlinear analysis. The frequency bands were defined as beta (13-21 Hz) and gamma (30-50 Hz) based on the results from previous studies.^1^,^2^,^31^

We used the Grassberger-Procaccia algorithm to calculate D2 from the EEG scalar time series *v(t_1_)(i= 1,2, …, N_t_)* where the index *i* represents the *i*-th sampled value of the total sampling number *N_t_*. The phase space can be reconstructed by using a sequence of *N=N_t_-(n-1)d* new vectors, *v_i_(i=1,…, N)=(v_i_, v_i+d_,……,v_i+(n-1)d_)* where d and n correspond to the delay number and embedding dimension, respectively. These vectors define the flow in the reconstructed n-dimensional phase space. The delay number, d, is determined by the first minimum of the mutual information function.^32^

This choice of d ensures that two successive delay coordinates are as independent as possible. Excessively short delays would compress the attractor to the diagonal of the reconstruction space, whereas for excessively large values of d, the structure of the attractor would disappear. The minimal embedding dimension, n, is determined by the procedure of Kennel et al.^33^

The correlation integral, C(*r*), represents the fraction of distances between the points on the attractor smaller than a certain distance, r:^34^-^36^


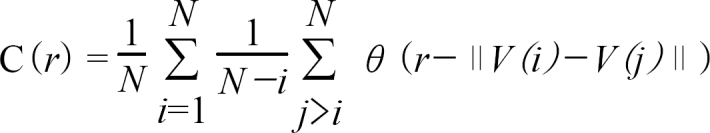


where *N* is the number of reference points and θ is the Heaviside function:


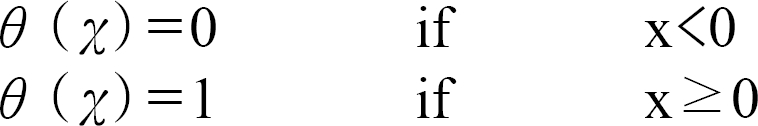


if N is sufficiently large, the following relation holds:


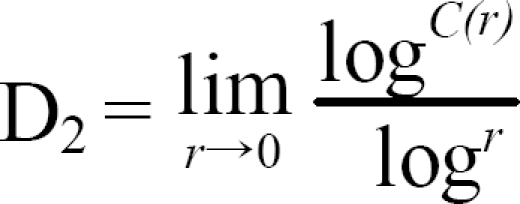


The dimension of the reconstructed attractor can be estimated from the slope of the linear region in the (log^*r*^, log^*C(r)*^) plot. This slope is determined by a linear regression of the (log^*r*^, log^*C(r)*^) plot between *r_min_* and *r_max_*, where *r_max_* and *r_min_* are chosen such that *C(r_max_)*=0.1 and *r_min_*=*r*_0_+0.5χ(*r_max_-r_0_*), with *r*_0_ being the smallest distance be-tween any two points on the attractor.

A delay number of 4 and an embedding dimension of 9 were used for both the AH and N-AH patients. The value of D2 was obtained by the commercial analysis program of Complexity ver.2.0 (LAXTHA Inc., South Korea). Group comparisons were performed with chisquare or independent t-tests.

## Results

The demographic data did not differ between the AH and N-AH patients except for the degree of education (Table 1). However, the educational level was not correlated with any of the dependent variables in either group (p>0.15 in all cases).

D2 did not differ between the two groups over the 1-70 Hz band. Bandpass filtering, however, revealed that D2 was higher in the AH patients than in the N-AH patients at Fp2 (2.09±0.39 vs. 1.83±0.49, p=0.045; Table 2) in the gamma band (30-50 Hz), and at P3 (2.11±0.27 vs. 2.30±0.27, p=0.018; Table 3) in the beta band (13-21 Hz).

Our results revealed that schizophrenia patients with AH have opposite DC values between the right prefrontal and left parietal cortex in their brain, which reflects the distinctive information processing in these regions.

## Discussion

The first main finding of our study was that the gamma frequency D2 in the right prefrontal cortex was higher in the AH patients than in the N-AH patients. The results from several studies support the notion that increased DC reflects active positive symptoms in schizophrenia patients. Koukkou et al.^37^ found that D2 under resting conditions was higher in first-episode acute schizophrenia patients than in healthy control subjects. Elbert et al.^38^ also found that the DC of the resting EEG was higher in inpatient schizophrenia patients than in control subjects at a frontal electrode position. Saito et al.^24^ reported increased anterior regional omega complexity in nine neuroleptic naive, first-episode acute schizophrenia patients. All of the patients in these studies had relatively recent onset and actively symptomatic schizophrenia. The findings of our study, involving subjects with persistent AH, were in line with the previous findings.^21^,^24^

Gamma oscillations (30-50 Hz) have been receiving more attention in research on cognitive processing, especially since it is now considered likely that gamma activity is involved in high-level cognitive processes.^39^ Gamma activity appears to be related to coherent object representations, and its role may extend to internally driven representations and the maintenance of information in memory.^40^ In addition, the results from previous studies suggest the importance of the prefrontal cortex in AH patients.^18^,^41^,^42^ This region is believed to be involved in executive functions that distinguish endogenous sensations from those arising from external influences.^17^,^18^,^41^ Thus, taking these two theories together, a chaotic integration of gamma frequency information in the prefrontal cortex in patients with AH could make them unable to discriminate internally generated sensory inputs from those of external origin. Another possibility is that increased D2 associated with right prefrontal activity is a secondary phenomenon related to the processing of prosodic and emotional responses,^42^,^43^ because AH in schizophrenia patients is typically derogatory and hostile in tone.

The second main finding of our study was that the beta frequency D2 in the left parietal brain region was lower in the AH patients than in the N-AH patients. Experimentally, beta frequency oscillations are generated following periods of synchronous gamma frequency activity.^8^,^44^,^45^ Comparable transitions from the gamma (30-50 Hz) to the beta 1 (12-20 Hz) range are seen in the human EEG in response to novel auditory stimuli.^6^ Functionally, the occurrence of subsequent beta oscillations might reflect the modification of the neuronal circuitry used to encode the sensory perception of auditory stimuli.^2^,^19^

Ropohl et al.^2^ reported an increase in the fast magnetoencephalographic activity (12.5-30 Hz) in the left auditory cortex in a schizophrenia patient who had persistent AH despite receiving the appropriate medication. Lee et al.^1^ also reported increased beta activity in the frontal and parietal cortex in AH patients. Haenschel et al.^6^ recorded the beta 1 (12-20 Hz) activity over the parietal cortex in 10 normal healthy subjects exposed to pure sinusoidal tones. These findings suggest that beta frequency activity is important in sensory encoding, regardless of its origin (i.e., internal or external).

Several studies have shown that DC decreases during active cognitive processing. Anokhin et al.^46^ revealed that DC was inversely correlated with EEG coherence during cognitive tasks. Kirsch et al.^22^ found that DC was lower in normal controls than in schizophrenia patients during cognitive tasks. These findings suggest that the integration of information in the brain during cognitive challenges becomes more coherent (less chaotic), possibly due to the attention being focused on the task at hand. Therefore, our findings of decreased beta frequency D2 in the parietal cortex might reflect active cognitive processing in this region during AH.

Even though our subjects had experienced treatment-refractory AH lasting for at least 2 years, we cannot be certain that hallucinations were occurring during the 30-s EEG epochs that were examined. Therefore, future nonlinear analysis studies should investigate any differences that may be present between the AH on and off states in the same subjects. Another limitation of the present study was the lack of healthy normal controls; comparisons with normal control subjects would produce more informative results, and this should be done in the future.

Our study implies the existence of a distinctive difference in DC between the right prefrontal and left parietal regions as the underlying biological correlate of AH in schizophrenia patients. We conclude that gamma frequency D2 in the right prefrontal cortex is more chaotic and beta frequency D2 in the left parietal cortex is more coherent (less chaotic) in AH patients than in N-AH patients.

## Figures and Tables

**TABLE 1:**
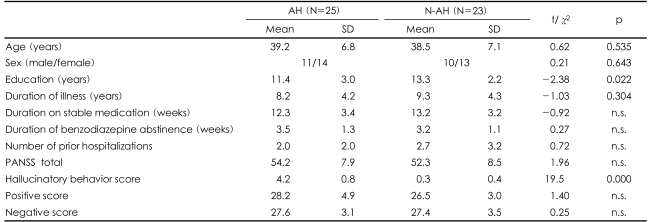
Demographic data and Positive And Negative Syndrome Scale (PANSS) scores of the auditory hallucination (AH) and non-AH (N-AH) patients

n.s.: not significant

**TABLE 2:**
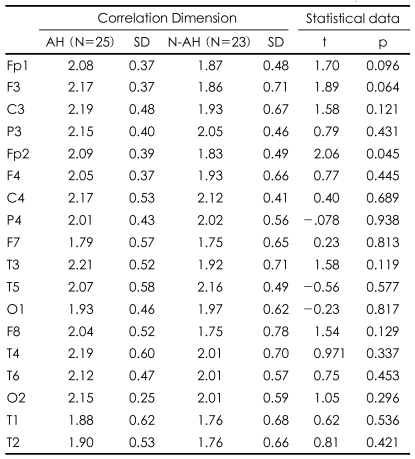
Gamma band (30-50 Hz) correlation dimension of 30-s resting electroencephalogram (EEG) epochs at each electrode in auditory hallucination (AH) and no AH (N-AH) patients

**TABLE 3:**
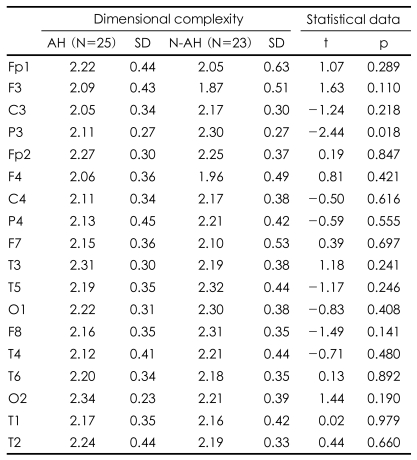
Beta band (13-21 Hz) dimensional complexity of 30-s resting electroencephalogram (EEG) epochs at each electrode in auditory hallucination (AH) and no AH (N-AH) patients
